# Marine Organisms as Potential Sources of Bioactive Peptides that Inhibit the Activity of Angiotensin I-Converting Enzyme: A Review

**DOI:** 10.3390/molecules24142541

**Published:** 2019-07-12

**Authors:** Dwi Yuli Pujiastuti, Muhamad Nur Ghoyatul Amin, Mochammad Amin Alamsjah, Jue-Liang Hsu

**Affiliations:** 1 Department of Marine, Faculty of Fisheries and Marine, Universitas Airlangga, Surabaya 60115, Indonesia; 2 Department of Biological Science and Technology, National Pingtung University of Science and Technology, Pingtung 91201, Taiwan; 3 Research Center for Austronesian Medicine and Agriculture, National Pingtung University of Science and Technology, Pingtung 91201, Taiwan

**Keywords:** ACE inhibitory peptide, antihypertensive, bioactive peptides, hypertension, marine resources

## Abstract

Angiotensin I-converting enzyme (ACE) is a paramount therapeutic target to treat hypertension. ACE inhibitory peptides derived from food protein sources are regarded as safer alternatives to synthetic antihypertensive drugs for treating hypertension. Recently, marine organisms have started being pursued as sources of potential ACE inhibitory peptides. Marine organisms such as fish, shellfish, seaweed, microalgae, molluscs, crustaceans, and cephalopods are rich sources of bioactive compounds because of their high-value metabolites with specific activities and promising health benefits. This review aims to summarize the studies on peptides from different marine organisms and focus on the potential ability of these peptides to inhibit ACE activity.

## 1. Introduction

Hypertension or high blood pressure is generally caused by behavioral risk factors, ageing, and population growth. It emerged in upper-middle income countries among adults aged >25 years. Hypertension causes 9.4 million deaths each year worldwide [1]. Currently, hypertension is one of the leading causes of morbidity and mortality globally, followed by metabolic disorder [2]. It is a key risk factor for cardiovascular disease, heart attack, stroke, and arteriosclerosis. The common examination used to diagnose hypertension is the measurement of blood pressure; a systolic blood pressure (SBP) and diastolic blood pressure (DBP) higher than 140 mm Hg and 90 mm Hg, respectively, indicates hypertension. To mitigate the aberrations in blood pressure and restore normal physiological function, functional molecules derived from food have been widely pursued.

The renin angiotensin aldosterone system (RAAS) plays a significant role in the maintenance of arterial blood pressure and fluid balance and is regarded as the major target to combat hypertension [3]. In RAAS, angiotensinogen is cleaved by renin, producing angiotensin I. Angiotensin I is then converted to angiotensin II, a strong vasoconstrictor, by angiotensin I-converting enzyme (ACE). In addition, ACE inactivates the vasodilator bradykinin, which acts as a mediator of inflammation, a natriuretic peptide, and a potent stimulator of vasodilator prostaglandins, and is involved in nitric oxide synthesis [4]. Because the production of angiotensin II increases blood pressure [5,6], the inhibition of ACE is a reliable strategy to control hypertension [7]. ACE inhibitors decrease ACE activity and indirectly reduce the angiotensin II level, thereby exerting a vasorelaxation effect on blood vessels [8]. Captopril, enalapril, lisinopril, and benazepril are commonly used as effective synthetic ACE inhibitors and have been developed for treating hypertension. However, synthetic drugs usually cause undesirable side effects [9,10]. To reduce these side effects, food-derived ACE inhibitory peptides are preferred over synthetic drugs to combat hypertension. ACE inhibitory peptides are considered as potent antihypertensive drugs, and they do not have any undesirable side effects. ACE inhibitors are more effective than other hypertensive drugs in retarding the progression of renal damage and reducing proteinuria. Two health organization, namely the international society of hypertension-world health organization (ISHWHO) and the Canadian society of hypertension recommend ACE inhibitors as the first line of treatment for hypertension [11]. 

Proteins are an important macronutrient as they provide the necessary energy and amino acids essential for growth and the maintenance of normal bodily functions. Many physiological and functional properties of proteins are attributed to bioactive peptides [8]. Bioactive peptides derived from food protein have been growing attractive because of awareness of their health-boosting properties. Bioactive peptides from several natural and processed foods have now been isolated and characterized. They function as potential physiological modulators in the process of metabolism during intestinal digestion and are liberated depending on their structure, composition, and amino acid sequence. Some bioactive peptides have been identified to possess nutraceutical potential and promote overall human health [12], with the potential of being used as candidates for treating conditions, such as hypertension [13].

Bioactive peptides are usually isolated from milk and cheese. They are also isolated from other animal sources, such as meat, gelatin, eggs, and various fish species (salmon, sardine, tuna, and herring), and plant sources, such as mushroom, wheat, pumpkin, and sorghum [14]. For example, ACE inhibitory peptides derived from fish have been shown to have a favorable effect on blood pressure [7,15,16]. Unlike many synthetic ACE inhibitors, which cause dry cough and angioedema, natural peptide-inhibitors have no side effects and are considered to be safer and healthier [17]. In recent years, ACE inhibitors have been derived from food proteins, such as milk [18,19], corn [20,21], ovalbumin [22], legume [23,24], Chinese soft-shelled turtle eggs [25,26], bitter melon seeds [27], cheese [28,29], chicken eggs [30,31,32,33], casein [34,35,36], fish [37,38,39], and algae [40,41].

Oceans cover >70% of the earth’s surface and are a rich resource for humans. There is increasing interest in marine organisms as new sources of natural products. Several compounds with unique biological activities have been isolated from marine organisms. The marine environment is rich in biological as well as chemical diversity; compounds isolated from marine organisms have been used as pharmaceuticals, nutraceuticals, cosmeceuticals, molecular probes, fine chemicals, and agrochemicals. Macro-and microorganisms in marine habitats possess a wide array of secondary metabolites, including terpenes, steroids, polyketides, peptides, alkaloids, polysaccharides, proteins, and porphyrins. Because the environment surrounding marine organisms is extreme, aggressive, and competitive, these organisms produce several secondary metabolites with a promising potential for use as drugs, nutritional supplements, and therapeutic agents [42,43,44]. Marine organisms, such as fish, shellfish, seaweed, microalgae, molluscs, crustaceans, and cephalopods, are rich sources of several functional compounds, such as bioactive peptides, enzymes, polyunsaturated fatty acids, vitamins, minerals, phenolic phlorotannins, and polysaccharides. Moreover, as some marine organisms, especially fish, are particularly rich sources of protein, they are ideal for generating protein-derived bioactive peptides [45,46]. Marine bioactive peptides have gained significant attention for their health promoting effects, such as antihypertensive, antioxidant, anticoagulant, antimicrobial, antithrombotic, and hypocholesterolemic properties [47]. Furthermore, compounds isolated from marine organisms have been commercially distributed in health markets [48]. In this review, we discuss the ACE inhibitory peptides derived from marine resources and provide information on their production, characterization, and potential health benefits. We also review the future prospects of ACE inhibitory peptides derived from marine organisms as therapeutic drugs to combat hypertension.

## 2. ACE Inhibitory Peptides Derived from Marine Organisms

**Figure 1:**
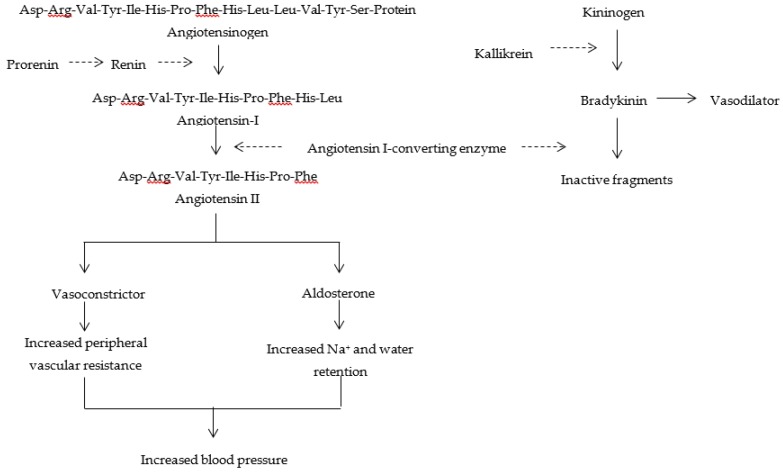
Role of angiotensin I-converting enzyme in the renin angiotensin aldosterone system and the kinin–kallikrein system [15].

Zinc ion (Zn^2+^)-dependent dipeptidyl carboxypeptidase, also known as ACE (EC 3.4.15.1), plays a pivotal role in the regulation of blood pressure because of its action in RAAS [49]. ACE is present in biological fluids, such as plasma and semen, and in many tissues, such as testis, intestinal epithelial cells, proximal renal tubular cells, brain, lungs, stimulated macrophages, vascular endothelium, and the medial and adventitial layers of blood vessel walls [4]. In humans, ACE exists in two isoforms: somatic ACE (sACE) and germinal ACE (gACE). sACE is distributed in many types of endothelial and epithelial cells, whereas gACE occurs in germinal cells in the testis, and is therefore also known as testicular ACE [6]. In RAAS, ACE cleaves the decapeptide angiotensin I (Asp-Arg-Val-Tyr-Ile-His-Pro-Phe-His-Leu) into the octapeptide angiotensin II (Asp-Arg-Val-Tyr-Ile-His-Pro-Phe) by removing the C-terminal dipeptide His-Leu. Angiotensin II stimulates the release of aldosterone and antidiuretic hormone or vasopressin, consequently increasing the retention of sodium and water; it also acts as a potent vasoconstrictor (Figure 1). These phenomena act in concert to directly increase the blood pressure [6]. Substrates of ACE include not only angiotensin I in RAAS and bradykinin in the kinin–kallikrein system, but also the haemoregulatory peptide *N*-acetyl-Ser-Asp-Lys-Pro, which is a putative bone marrow suppressor. It contributes to haemopoietic cell differentiation, regulating tissue and blood levels of the vasoactive hormones angiotensin II and bradykinin [50]. In addition, ACE shows endopeptidase activity against a wide range of substrates, such as cholecystokinin, substance P, and luliberin. The inhibition of ACE enzymatic activity on angiotensin I is one of the major challenges to combat hypertension-related disorders [51].

Recently, natural marine products have been investigated as alternative synthetic drugs; they have been the topic of interest for many researchers due to their numerous beneficial effects, and some novel ACE-inhibitory compounds have been isolated from algae [52,53]. Marine proteins, such as Heshiko, a fermented mackerel product [38], sardine muscle [9], shark meat [54], Alaska pollock skin [55], marine shrimp [56], and chum salmon [57], exhibit ACE inhibitory activity. ACE inhibitory peptides usually contains 2–12 amino acid residues [10,58,59]. However, some studies have identified up to 27 amino acid residues in ACE inhibitory peptides [60,61]. Proteases, such as pepsin, chymotrypsin, alcalase, and trypsin, are frequently used in hydrolysis for generating ACE inhibitory peptides [9,10,55]. List of identified peptides derived from marine resources; origin, sequence peptides, and IC_50_ value, can be seen in Table 1.

The potency of peptides derived from marine organisms is expressed as the half maximal inhibitory concentration (IC_50_), which indicates the ACE inhibitor concentration that leads to 50% inhibition of ACE activity. Moreover, Lineweaver–Burk plots are usually used to determine the inhibition mode of ACE inhibitory peptides. Most of the reported peptides act as competitive inhibitors of ACE. In the competitive inhibition mode, the inhibitor competes with the substrate and binds to the active site of ACE. In the non-competitive inhibition mode, the inhibitor binds to a site other than the active site. The binding of inhibitor to ACE alters the conformation of ACE, which prevents the substrate from binding to the active site of ACE. The enzyme, substrate, and inhibitor cannot form a complex; thus, the enzyme–substrate complex or enzyme–inhibitor complex is formed. In the uncompetitive inhibition mode, the inhibitor binds to only the substrate–enzyme complex. The C-terminal end of the inhibitory peptide associates with the active site pockets of ACE. ACE harbors three sub-sites: antepenultimate position (S1), penultimate position (S1′), and ultimate position (S2′). In the substrate, the amino acids Pro, Ala, Val, and Leu are the most favorable for S1; Ile is the most favorable for S1′; and Pro and Leu are the most favorable for S2′ [77]. The S1 sub-site includes Ala354, Glu384, and Tyr523 residues; S1′ pocket contains Glu162; and S2′ pocket includes Gln281, His353, His513, Lys511, and Tyr520 [78,79]. Many studies have shown that peptides with high ACE inhibitory activity contain Trp, Phe, Tyr, or Pro at the C-terminus and branched aliphatic amino acids at the N-terminus [49].

In China, soft-shelled turtle eggs have been used as a tonic food for a long time. Low-molecular weight peptides (<3 kDa) have been isolated from soft-shelled turtle egg by ultrafiltration and fractionated by reversed-phase high-performance liquid chromatography (RP-HPLC). In vitro screening of the resulting fractions for ACE inhibitory activity has revealed an IC_50_ value of 4.39 µM for the peptide IVRDPNGMGAW isolated from soft-shelled turtle egg white. This peptide has been identified as a competitive inhibitor of ACE [26]. The peptide AKLPSW, isolated from soft-shelled turtle egg yolk, has also been shown to exhibit potent ACE inhibitory activity, with an IC_50_ value of 15.3 µM, and inhibition kinetics has indicated that this peptide is a non-competitive inhibitor of ACE. The AKLPSW peptide significantly reduces the systolic blood pressure by approximately 13 mm Hg after 6 h of oral administration, thus confirming its antihypertensive effect [25]. In another study, Sardinella protein hydrolysates (SPHs) were obtained from fermentation with *Bacillus subtilis* (SPH-A26) and *Bacillus amyloliquefaciens* (SPH-An6). Approximately 800 peptides have been identified in SPH-A26 and SPH-An6 using nano electrospray ionization liquid chromatography tandem mass spectrometry. Of these 800 peptides, eight isolated from SPH-A26 and seven from SPH-An6 have been selected based on homologies with previously characterized peptides (Biopep data bank), as well as peptide length. Among the synthesized peptides, NVPVYEGY and ITALAPSTM show ACE inhibitory activity with IC_50_ values of 210 and 229 µM, respectively. Fermented SPHs have a potential for use as hypotensive nutraceutical ingredients [80]. The popular freshwater tilapia also reported the potential antihypertensive peptides from hydrolysate by using papain, bromelain, and pepsin. In order to enhance the activity, the hydrolysate was fractionated into four fractions (<1 kDa, 1–3 kDa, 3–5 kDa, and 5–10 kDa). The pepsin-hydrolyzed FPH (FPHPe) with the highest DH (23%) possessed the strongest ACE-inhibitory activity (IC_50_ of 0.57 mg/mL). Its <1 kDa ultrafiltration fraction (FPHPe1) suppressed both ACE (IC_50_ of 0.41 mg/mL). In addition, FPHPe1 significantly reduced SBP (maximum −33 mmHg), DBP (maximum −24 mmHg), mean arterial pressure (MAP) (maximum −28 mmHg), and hearth rates (HR) (maximum −58 beats) in SHRs [81]. 

The production of peptides with ACE inhibitory activity must consider the amino acid composition and molecular weight of hydrolysates. Purification is carried out to obtain a single peptide with a specific amino acid residues which is in accordance with characterized sequence of bioactive peptide inhibiting ACE. The pure peptide could be easily observed its activity and stability, as well as the dosage of peptide administration in the patients with hypertension symptom would be validly determined. Total hydrolysates with high molecular weight revealed lower activity for inhibiting the ACE rather than single peptide. The shorter amino acid residues is more visible to reach the target site when through the digestive tract and they can be absorbed easily. Then, lower-molecular weight peptides also have a higher probability of passing through the intestinal barrier and exerting biological function [65]. The C-terminal residue in tripeptides or dipeptides plays an important role in binding to sub-sites S1, S1′, and S2′ sub-sites within the active site of ACE [82]. Aromatic or hydrophobic amino acid residues, such as Trp, Phe, Tyr, and Pro, are more active if present at positions in the C-terminal end that bind to each of the three sub-sites of ACE. In addition, tripeptides or dipeptides with a branched aliphatic amino acid at the N-terminus show potent ACE inhibition. Basic amino acid residues, such as Lys and Arg, at the C-terminus also contribute to potent inhibition against ACE [83]. Many studies have shown that the C-terminal residue of potent ACE inhibitory peptides is usually a hydrophobic amino acid [39,70,74,84,85].

There is no correlation between competitive inhibitor with high ACE inhibitory activity. Several non-competitive inhibitors show high ACE inhibitory activity. The peptide Ala-Lys-Leu-Pro-Ser-Trp derived from soft-shelled turtle egg yolk exhibits a low IC_50_ value of 13.7 µM [25], whereas the peptide Val-Glu-Leu-Tyr-Pro isolated from cuttlefish muscle protein exhibits an even lower IC_50_ value of 5.22 µM [74]; both these peptides are considered non-competitive inhibitors. Moreover, some peptides inhibit ACE activity by the uncompetitive mode of inhibition. For example, the peptides Ile-Trp and Phe-Tyr have been ientified as uncompetitive inhibitors [86]; similarly, the peptides Tyr-Ley-Tyr-Glu-Ile-Ala and Tyr-Leu-Tyr-Glu-Ile-Ala-Arg-Arg have been identified as uncompetitive inhibitors [87]. Depending on the results of pre-incubation of the peptide with ACE, the ACE inhibitory peptides are divided into three categories: true inhibitors, prodrugs, and real substrates. A true inhibitor shows no significant difference in the IC_50_ value before and after pre-incubation with ACE, whereas a prodrug shows dramatic reduction in the IC_50_ value after pre-incubation with ACE. On the other hand, a real substrate shows an increase in the IC_50_ value after pre-incubation with ACE, suggesting a reduction in its inhibitory activity against ACE. Generally, the prodrug- and true inhibitor-type peptides are expected to exhibit long-lasting antihypertensive activity in spontaneously hypertensive rats used as a model to study hypertension in humans [88,89]. 

## 3. Generation of Bioactive Peptides

Protein hydrolysates have an excellent amino acid balance, are readily digestible, show rapid uptake, and contain bioactive peptides [90]. Bioactive peptides act as therapeutic agents and are characterized by high biological specificity, low toxicity, high structural diversity, high and wide spectrum of activity, and small size, which implies that they have a low likelihood of triggering undesirable immune responses [91]. Bioactive peptides are defined as protein fragments with beneficial effects on bodily functions and human health. Peptides isolated from food sources are structurally similar to endogenous peptides and therefore interact with the same receptors and play a prominent role as immune regulators, growth factors, and modifiers of food intake [92]. Depending on the sequence of amino acids, these peptides can exhibit diverse activities, including antimicrobial [93], antioxidant [94], antithrombotic [95], and antihypertensive [25]. 

Bioactive peptides are generally produced via enzymatic hydrolysis using digestive enzymes, fermentation using proteolytic starter cultures, or proteolysis using microorganism-or plant-derived enzymes. To generate short-chain functional peptides, enzymatic hydrolysis is used in combination with fermentation or proteolysis [96]. During growth, microorganisms release the protease enzyme into the extracellular medium, leading to proteolysis and peptide generation. Microorganisms are typically used for fermentation for several hours to several days, depending on the desired peptide and the type of fermentation [97]. During fermentation, microorganisms break down complex compounds into smaller molecules with various physiological functions [98]. Fermented marine food products are rich sources of bioactive compounds, including amino acids and peptides [99]. Digestive enzymes, such as trypsin, chymotrypsin, and pepsin, release the bioactive peptides for gastrointestinal digestion in vivo. To stimulate gastrointestinal digestion, several proteolytic enzymes, such as alcalase and thermolysin, engage with trypsin and pepsin. In addition, recombinant DNA technology and chemical synthesis have been used to produce bioactive peptides [92]. The physicochemical properties, such as molecular weight, isoelectric point, and hydrophilic or hydrophobic indices of the resulting peptides, change after enzymatic hydrolysis. Prominent amino acids, such as Pro and Val, play key roles in most antihypertensive peptides [91].

In the digestive system, bioactive peptides are absorbed through the intestine and enter the blood stream to exert systemic effects or local effects in the gastrointestinal tract. Dipeptides and tripeptides are easily absorbed in the intestine. To exert antihypertensive effects, bioactive peptides must reach the target cells after absorption through the intestine. Common bioactive peptides with antihypertensive effects include Val-Pro-Pro (VPP) and Ile-Pro-Pro (IPP); they are produced via fermentation using *Lactobacillus helveticus* and *Saccharomyces cerevisiae*. These two peptides have been detected in the aortal tissue using HPLC, and their effect on ACE activity was lower in the aorta in the study group than in the control group (saline) [14].

## 4. Screening Approach

The search for peptides capable of inhibiting ACE activity has been intensified. The pursue of ACE inhibitory peptides from marine, as well as other sources, has been substantiated. A reliable assay to determine the ability of peptides to inhibit ACE activity is of paramount concern. In vitro determination of ACE inhibitory peptides is preceded by enzymatic digestion or microbial fermentation, followed by the analysis of structure and chemical synthesis of active peptides. Most assays evaluating the ACE inhibitory activity of peptides have been performed as described previously [100]. The technique used to evaluate the ACE inhibitory activity of peptides must be simple, sensitive, and reliable. Several such methods have been developed, such as spectrophotometry, HPLC, fluorometric capillary electrophoresis, and radiochemistry. Among these, spectrophotometry is the most commonly used method to measure ACE inhibitory activity. This method involves the hydrolysis of hippuryl-histidyl-leucine (HHL) by ACE to hippuric acid (HA). The amount of HA produced from HHL is directly correlated with ACE activity [101]. The amount of HA formed is determined by measuring the absorbance at 228 nm (absorption maximum of HA) [102]. Although the spectrophotometry is useful, it is time consuming, complicated, and is unable to detect trace amounts of the sample.

In practice, results of different assays may vary because of the use of different substrates, such as the synthetic peptides HHL and furanacryloyl-l-phenylalanylglycyl-glycine (FAPGG), which are the most commonly used substrates, and the fluorescent molecule o-aminobenzoylglycyl-_P_-nitrophenylalanylproline for specific detection and quantification [103]. Results may also vary within the same assay because of the use of different test conditions or the use of ACE from different origins. Thus, ACE activity levels must be carefully controlled to obtain comparable and reproducible results [83,104].

HPLC is a common method to determine ACE inhibitory activity of peptides as it generates reproducible results. Although HPLC has been used for decades, it requires the extraction of the product from the reaction mixture using an organic solvent, which limits the number of samples that can be analyzed per day and is also a source of error [105]. Moreover, HPLC analysis shows peculiar results from samples with added inhibitor, which exhibit high HA release than samples without the added inhibitor. This occurs if the enzyme or the substrate (HHL) is unstable in solution. The evaluation of ACE inhibition is depends on the comparison between the concentration of HA in the presence or absence of an inhibitor (inhibitor blank). The occurrence of autolysis of HHL to give HA was evaluated by a reaction blank, i.e., a sample with the higher inhibitor concentration and without the enzyme [24]. Another substrate, FAPGG, has also been used for HPLC [106,107]; FAPGG releases 2-furylacryloyl-l-phenylalanine (FAP) as a product. This method is used to quantitate the levels and can be used a model of inhibition according to the sigmoid character of the response curve. The slope of the curve, describing absorbance versus time, is thus a direct measure of ACE activity. It is based on the combination of enzymatic reaction with HPLC detection of the inhibition of enzyme activity by measuring the levels of the substrate and product formed. The amount of FAP formed is determined by measuring the absorbance at 305 nm. This method is beneficial, as it does not require sophisticated equipment or radiolabelled compounds [108]. Because the price of the two substrates, HHL and FAPGG, is similar, the HPLC method is advantageous over spectrophotometry, as it requires less labor and has a higher throughput than spectrophotometry [103].

The determination of ACE activity also utilizes fluorescent tripeptides, such as *o-*aminobenzoylglycyl-*p*-nitro-l**-**phenylalanyl-l-proline [Abz-Gly-Phe(NO_2_)-Pro]. The hydrolysis of this substrate by ACE generates o-aminobenzoylglycine (Abz-Gly) as a product, which is easily quantified fluorometrically using appropriate excitation and emission wavelengths. Fluorescence detection of the reaction products is highly sensitive and precise. Moreover, commercial availability of all reagents is a major advantage, allowing easy introduction of the assay in laboratories [109].

**Figure 2:**
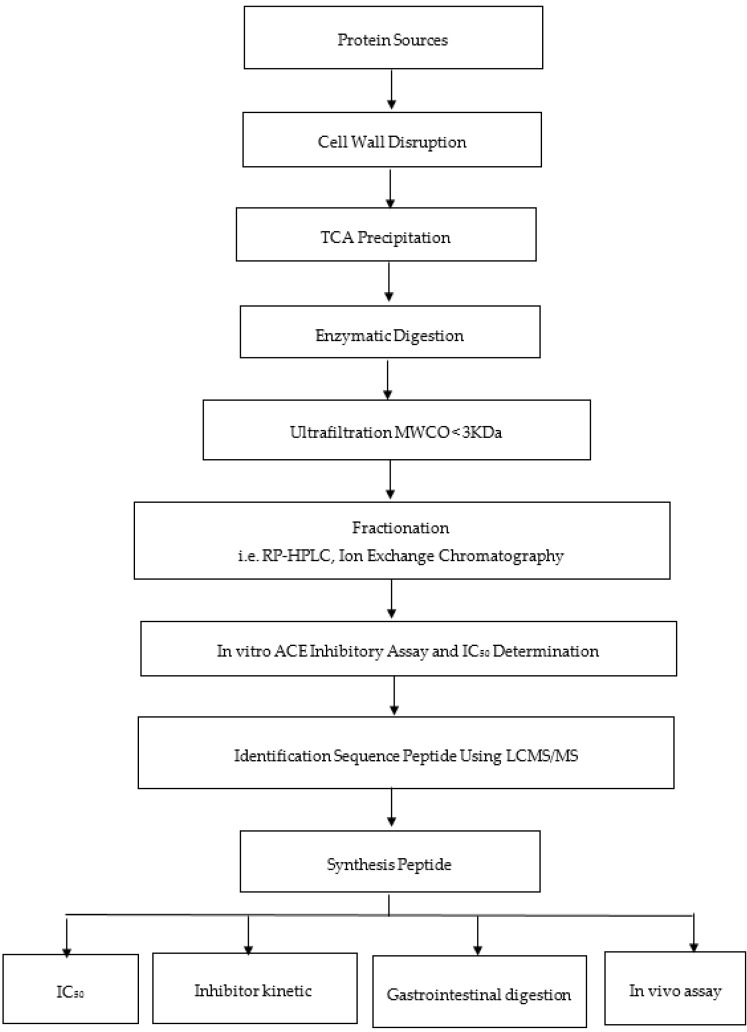
Flowchart showing the production of bioactive peptides for angiotensin I-converting enzyme (ACE) inhibitory assay [25].

To obtain ACE inhibitory peptides, slight modification of the assay is crucial. Orthogonal bioassay-guided fractionation is considered as a potential method to obtain ACE inhibitory peptides. This method involves the separation of the potential peptides using two ways of fractionation: Strong cation exchange (SCX) and RP-HPLC (Figure 2). SCX separates peptides based on their charge, whereas RP-HPLC separates peptides based on their hydrophobicity [110]. Although both SCX and RP-HPLC separate peptides using different mechanisms, peptides are regarded as potential ACE inhibitors because they remain in the most active fraction using both methods. Pujiastuti et al. [25] revealed the identification of overlapping peptides using SCX and RP-HPLC.

A new method used to measure ACE activity is ultra-performance liquid chromatography (UPLC). The UPLC-mass spectrometry method has been developed to determine ACE activity using HHL as the substrate and purified rabbit ACE. This method is rapid, accurate, and reproducible, and is used to determine trace amounts of compounds. In addition, this method requires a short analysis time and small reaction volume and is highly selective compared to conventional methods. It is also suitable for high-throughput screening of potential ACE inhibitors and candidate compounds isolated from herbal medicines [111].

The in vitro gastrointestinal digestion approach provides a straightforward approach to imitate peptide function by incubating the peptide with ACE before in vivo oral administration. Oral administration of ACE inhibitory peptides in hypertensive patients requires these peptides to pass through the digestive tract and be absorbed through the intestinal epithelium. Pepsin is widely used to represent gastrointestinal enzymes that function at acidic pH. Polypeptides are further truncated by pancreatic proteases, including trypsin, α-chymotrypsin, elastase, and carboxypeptidases A and B at alkaline pH. In vivo testing of peptides is frequently performed in spontaneously hypertensive rats as they mimic hypertension in humans. This animal model has been used to evaluate the effects of both short-and long-term administration of antihypertensive peptides. In human studies, food-derived peptides have been used to establish whether peptides exhibit an antihypertensive effect in humans with high-to-normal blood pressure. For example, the antihypertensive effect of the peptides IPP and VPP isolated from the commercial fermented milk show antihypertensive effects after long-term administration. The sour milk product Calpis from Japan has been examined in mildly hypertensive patients [112]. In some cases, ACE inhibitory peptides fail to show hypotensive activity after oral administration in vivo, possibly because of the hydrolysis of these peptides by ACE or gastrointestinal proteases [74,113]. It is difficult to evaluate a direct correlation between in vitro ACE inhibitory activity and in vivo antihypertensive activity because the bioavailability of these peptides after oral administration varies. ACE inhibitory peptides must remain active during gastrointestinal digestion and reach the specific organ. However, it is possible that ACE inhibitory peptides are degraded before reaching the specific organ. The antihypertensive mechanism of ACE inhibitory peptides, rather than the ACE inhibition mechanism, may be of greater interest [77,114].

In silico methods are used to predict the structure of ACE inhibitory peptides based on similarity between sequences available in databases. The molecular docking approach is widely used to predict and characterize the binding site of target proteins according to ligand conformation and binding affinity score [115]. The most convenient approach to elucidate the accuracy of molecular docking is to determine the distance of binding conformation using the scoring function in the docking program [116]. Several scoring functions are used to evaluate the docking procedure, such as CDocker Energy, CDocker Interaction Energy, LibDockScore, PLP1, PLP2, LigScore1, LigScore2, Jain, PMF, and PMFO4. Besides, BIOPEP-UWM and BLAST database is increasingly popular to be in silico approaches for investigating biological activities from tilapia and chickpea [117]. BIOPEP-UWM database is used to predict bioactive peptides composed in protein sequences. This method has benefits such as time and cost reduction, as well as being a rapid method to identify and characterize proteins. Briefly, the bioactivities, sequences, number, and location of the peptides were obtained from the sequences of the identified proteins analyzed using the “profiles of potential bioactivity” tool. Moreover, the sequences of the identified proteins were examined using the “enzyme action” tool to simulate enzymatic hydrolysis [118]. Knowing the position of the binding site before docking significantly increases the docking efficiency. Moreover, knowledge of the structure and activity relationship is important to explore potential ACE inhibitory peptides. The ACE structure contains a Zn site, which usually coordinates with oxygen, nitrogen, and sulphur donors of Asp, Cys, and His, respectively, wherein His is the most regularly encountered in the sphere of Zn^2+^ ion. The other Zn ligand in catalytic sites is water; it is activated for polarization, ionization, and arrangement of ligands in coordination with Zn [11]. The Zn^2+^ ion is also important for the binding strength between ACE and its inhibitors [119]. Generally, ACE inhibitors contain one or more molecular functionalities, such as Zn-binding ligand, a hydrogen bond donor, and a carboxyl-terminal group [120]. The ability of a protein to interact with small molecules plays a major role in the dynamics of that protein, which may enhance or inhibit its biological function. Studies on the catalytic mechanism of ACE have revealed that the 19 amino acid residues in the active site of ACE, including His353, Ala354, Ser355, Ala356, His383, Glu384, His387, Phe391, Pro407, His410, Glu411, Phe512, His513, Ser516, Ser517, Val518, Pro519, Arg522, and Tyr523, bind to small molecules or to protein (ligand).

## 5. Conclusions

Bioactive peptides derived from marine resources have potential ACE inhibitory activity and are considered as therapeutic agents to combat hypertension. The main characteristic of ACE inhibitory peptides is the position of the hydrophobic residue, usually Pro, at the C-terminus. In vitro and in vivo testing are the most challenging tasks in antihypertensive research as their results do not always show direct correlation, although gastrointestinal digestion is suggested to mimic peptide release in human body. Marine organisms represent sustainable sources of ACE inhibitory peptides for the production of pharmaceuticals and nutraceuticals at an industrial scale. Due to the importance of pure peptide inhibiting ACE for future pharmaceutical and nutraceutical industry, the purification techniques of identified peptide is highly crucial. Therefore, upscaling research on bioactive peptide purification should trigger biotechnologists to perform the research.

**Highlights:**

- Angiotensin I-converting enzyme (ACE) is a key target for treating hypertension.
- Food-derived bioactive peptides inhibit ACE activity, decreasing blood pressure.
- These peptides improve bodily functions and human health, without adverse effects.
- Marine organisms are sustainable sources of ACE inhibitory peptides.
- Various methods for their industrial production and testing are available.

## Figures and Tables

**Table 1:** List of identified peptides derived from marine resources; origin, sequence peptides, and IC_50_ value.

Origin	Enzyme	Sequence Peptide	IC_50_ (µM)	Reference
**Fish**				
Sea bream	Alkaline Protease	GY	265	[62]
		VY	16	
		GF	708	
		VIY	7.5	
Lizard fish	Neutral Protease	MKCAF	45.7	[63]
		RVCLP	175	[64]
Alaska pollock (*Theragra chalcogramma*)	Alcalase, Pronase E and Collagenase	GPL	2.6	[55]
		GPM	17.3	
Grass carp	Alcalase	VAP	19.9	[10]
Atlantic salmon (*Salmo salar* L.)	Alcalase and Papain	AP	356.9	[65]
		VR	1301.1	
Skipjack (*Katsuwonus pelamis*)	Alcalase	DLDLRKDLYAN	67.4	[66]
		MCYPAST	58.7	
		MLVFAV	3.07	
Yellowfin sole (*Limanda aspera*)	Chymotrypsin	MIFPGAGGPEL	268.3	[67]
Pacific cod	Pepsin	GASSGMPG	6.9	[68]
		LAYA	14.5	
*Paralichthys alivaceus*	Pepsin	MEVFVP	79	[69]
		VSQLTR	105	
*Channa striatus*	Thermolysin	VPAAPPK	0.45	[70]
		NGTWFEPP	0.63	
**Microalgae**				
Chlorella vulgaris	Pepsin	IVVE	315	[40]
		FAL	26.3	
		AEL	57.1	
		VVPPA	79.5	
		AFL	63.8	
Chlorella ellipsoidea	Alcalase	VEGY	128.4	[71]
Spirulina platensis	Pepsin	IAE	34.7	[40]
		IAPG	11.4	
		VAF	35.8	
**Molluscs**				
Sea cucumber (*Acaudina molpadioidea*)	Bromelain and Alcalase	MEGAQEAQGD	15.9	[72]
Cuttlefish (*Sepia officinalis*)	Cuttlefish hepatopancreas	VYAP	6.1	[73]
		VIIF	8.7	
		MAW	16.32	
		GIHETTY	25.66	[74]
		EKSYELP	14.41	
		VELYP	5.22	
Squid *(Dosidicus gigas*) skin collagen	Esperase	GRGSVPAPGP	47.78	[75]
*Corbicula fluminea*	Protamex + Flavourzyme	VKP	3.7	[76]
		VKK	1045	
